# The effects of *NLRP3* rs10754558 and rs4612666 polymorphisms on preeclampsia susceptibility, onset, and severity: a case-control study and in silico analysis

**DOI:** 10.22099/mbrc.2024.49510.1936

**Published:** 2024

**Authors:** Mahnaz Rezaei, Marzieh Ghasemi, Mohsen Saravani, Rahele Ghasemian- Moghadam, Hossein Shahraki-Ghadimi, Mahtab Norouzi, Saeedeh Salimi

**Affiliations:** 1 Cellular and Molecular Research Center, Resistant Tuberculosis Institute, Zahedan University of Medical Sciences, Zahedan, Iran; 2 Department of Clinical Biochemistry, School of Medicine, Zahedan University of Medical Sciences, Zahedan, Iran; 3 Department of Obstetrics and Gynecology, Pregnancy Health Research Center, Zahedan University of Medical Sciences, Zahedan, Iran; 4 Pregnancy Health Research Center, Zahedan University of Medical Sciences, Zahedan, Iran; 5 Department of Clinical Biochemistry, School of Medicine, Shahid Beheshti University of Medical Sciences, Tehran, Iran

**Keywords:** NLRP3, Onset, Polymorphism, Preeclampsia, Severity

## Abstract

Preeclampsia (PE) is one of the serious complications of pregnancy and its exact etiology is unknown. Inflammasomes are multiportion complexes whose relation with PE has been described. Evidence showed the effect of NLRP3 inflammasome in PE pathogenesis. In the current study, we investigated the possible impacts of *NLRP3* polymorphisms on PE. A total of 252 PE and 258 control pregnant women were selected for the study. The PCR-RFLP method was employed to genotype rs10754558 and rs4612666 polymorphisms. The RNAsnp and SpliceAid 2 software were used for in silico analysis. There was no relationship between *NLRP3 *polymorphisms and PE. In comparison to control women, the *NLRP3* rs10754558 could increase the risk of severe PE in codominant and dominant models (OR=1.89, 95% CI=1.19-3.01, P=0.012, OR=1.95, 95% CI=1.24-3.06, P=0.0037, respectively). The findings of the in silico analysis revealed the effects of rs10754558 C to G and rs4612666 C to T substitutions on protein binding sites and rs10754558 C to G substitution on secondary RNA structure. These findings could confirm the finding those studies reported the impacts of these variants on various diseases. In conclusion, the *NLRP3* rs10754558 variant was associated with an increased risk of EOPE and severe PE.

## INTRODUCTION

Preeclampsia (PE) is considered the major cause of pregnancy-related death which includes between 2 and 8% of pregnancies [1]. The prevalence of PE has been estimated near to 5% in Iran [2]. This multi-system complication causes many problems in both the mother and the fetus. Maternal complications could include thrombocytopenia (a condition in which you have a low blood platelet count), dysfunctional liver, MAHA (signifying microangiopathic hemolytic anemia), acute kidney injury (AKI), placental abruption, visual disturbances, stroke, seizures, and pregnancy-related death. The fetus faces the risk of intrauterine growth restriction and prematurity[1]. This disorder is defined by a high blood pressure increase (*≥*140/*≥*90 mmHg) and proteinuria (*≥*0,3 gr/24 h) following the 20th week of gestation [3]. 

The insufficient depth of placental cytotrophoblast, defective trophoblast invasion, and insufficient maternal spiral artery remodeling in response to placental hypoxia and ischemia were introduced as the main causes of PE. In addition, the role of several molecular mechanisms, including altered angiogenic balance, systemic inflammation, and autophagy in the pathogenesis of preeclampsia has been identified [4]. 

One of the serious reasons for PE can be mentioned as an excessive MSIR (standing for maternal systemic inflammatory response) to the gestation. The response is stimulated by the activation of both the innate immune system and the adaptive immune system. PE is a condition with chronic inflammation by activation of B cells, APCs ( standing for antigen-presenting cells), the T helper cells (Th cells), and natural killer cells, also known as NK cells which can contribute to the occurrence of PE symptoms during gestation [5].

Inflammasomes are considered multiprotein and self-organizing complexes occurring in the cytoplasm of the cells of the innate immune system that not only contribute to both the activation of inflammatory response and the production of IL-18 and IL-1β but also act like well-tuned alarm system including a sensor molecule, the AP ( signifying adaptor protein) and the pro-inflammatory caspase-1[6]. PAMPs (standing for pathogen-associated molecular patterns) or endogenous DAMPs (representing danger signals/alarmins/damage-associated molecule patterns) are done by the sensor molecule [6]. Then the occurrence of both the formation of the inflammasome complex and the stimulation of ca[spase 1 leads to initiating downstream responses, such as the processing and release of IL-1b and IL-18, as well as pyroptosis, which can be considered as lytic cell death [7].

Initially, inflammasomes were considered to be specific to the signaling pathway of innate immune, but new investigations have indicated that these platforms also play a role in enhancing adaptive immune responses. So far, the properties of five distinct inflammasomes are precisely known, each recognized by its specific sensor molecule [8]. 

The best-known inflammasome is the NLRP3 inflammasome which is distinguished by two main definitions [9]. First, various structurally unrelated molecules can activate this complex [10]. Second, the NLRP3 is expressed at high levels in innate immune cells [11]. Given that the components of inflammasomes are expressed by the cells from the placenta, preliminary research has demonstrated that inflammasomes play a role in the responses to inflammation related to placental disorders [8].

Mulla et al. and Xie et al demonstrated that NLRP3 activated in both the trophoblasts, which are specialized cells of the placenta and peripheral blood was involved in the PE development [12, 13]. Descriptive research also found that the placental IL-1b, caspase-1, and NLRP3 expression in women diagnosed with severe PE were greater than those in normotensive pregnant women [14]. A new investigation with mouse models and human tissue revealed that extracellular vesicles derived from endothelium could stimulate the NLRP3 inflammasomes, leading to the activation of preeclampsia-like syndrome. Therefore, these data indicated that the NLRP3 activation in placental inflammatory processes is associated with the PE pathophysiology [8].

Several polymorphisms in the *NLRP3* gene have been described and their effects on various diseases like immune-inflammatory diseases have been reported [14]. The possible effects of *NLRP3* rs10754558 and rs2027432 polymorphisms on PE have been investigated in two previous studies [15, 16]. Therefore, in the present study, we investigated the impacts of *NLRP3 *rs10754558 and rs4612666 polymorphisms on the susceptibility, severity, and onset of PE.

## MATERIALS AND METHODS

**The study participants:** In this case-control study, 258 healthy and 252 PE pregnant women in the age range of 25 to 35 years referred to the affiliated Hospital of Zahedan University of Medical Sciences, Iran, were included. The Ethical Committee of Zahedan University of Medical Sciences approved the study protocol, and the study protocols and objectives were explained to all participants. The PE group was selected according to SBP (standing for systolic blood pressure) ≥140 mmHg and DBP (signifying diastolic blood pressure) ≥ 90 mmHg on two or more occasions at least 6 h apart as well as the proteinuria detected by urine dipstick test (≥0.3 g/24hr or ≥+1) following 20 weeks of gestation. Participants who had the following characteristics, such as hydrops fetal, twin or multiple pregnancies, and each systemic disease were excluded from the study. Severe PE was diagnosed with chronic high blood pressure (SBP ≥160 mmHg or DBP ≥110 mmHg) or high proteinuria (≥5 g protein in a 24-hour urine collection) on two occasions at least 4 hours apart [3]. It is also defined as the absence of new-onset hypertension, proteinuria, thrombocytopenia, liver function which is impaired, poor function of the kidney, pulmonary edema, then the onset of new headache, and visual disturbances which can be defined as anything that impacts the ability to see clearly and comfortably. Early-onset and Late-onset PE are described as the diagnosis of PE before and after the 34th week of gestation respectively. The inclusion and exclusion criteria for this study were determined by our prior study [17].

**Genetic analysis:** Two ml of blood was taken from all subjects, and then all samples were placed in a freezer at -20°C. For DNA extraction the salting-out method was done. The PCR-RFLP analysis was employed to genotype *NLRP3* rs10754558 and rs4612666 variants, as described previously [18, 19]. 

**Statistical analysis: **The data were analyzed by using SPSS version 23. The differences between two groups were assayed with independent samples t-test and chi-square tests whenever suitable. Using SNPStats (https://www.snpstats.net/start.htm) the genotypic and allelic differences between the PE and control groups as well as their subgroups were determined. If the p-value was less than 0.05, it is considered as significant. 

**In silico analysis: **For in silico analysis, two online tools, namely RNAsnp and SpliceAid 2 were used [20, 21]. RNAsnp is a tool that can be used to investigate changes in the secondary structure of RNA under the influence of snp. Using the SpliceAid 2 tool, the effects of SNPs on the binding sites of regulatory proteins during transcription can be studied.

## RESULTS

Table S1 presents the clinical and general data of the PE group and controls. The allele and genotype frequencies of maternal *NLRP3* variants in the PE group and controls are shown in Table 1. Both loci were in Hardy-Weiberg equilibrium. There was no relationship between rs10754558 and rs4612666 polymorphisms and the susceptibility of PE in any genetic models. The results obtained from haplotype analysis demonstrated that the frequency of four haplotypes of rs10754558 and rs4612666 variants did not differ between the two groups (Table 2).

As shown in Table S2, the *NLRP3* rs10754558 variant could enhance the risk of EOPE in the PE group only in the dominant model as compared with controls (OR=1.74, 95% CI=1.05-2.87, P=0.032). 

Table S3 shows the frequencies of *NLRP3* variants in patients diagnosed with severe and mild PE and the controls. The *NLRP3* rs10754558 could enhance the risk of severe PE in comparison with that of mild PE in the codominant (OR=2.07, 95% CI=1.21-3.54, P=0.0048) and dominant models (OR=2.22, 95% CI=1.31-3.76, P=0.0027). Indeed, the *NLRP3* rs10754558 was linked to an increased risk of severe PE in comparison to controls in codominant (OR=1.89, 95% CI=1.19-3.01, P=0.012) and dominant models (OR=1.95, 95% CI=1.24-3.06, P=0.0037). Therefore, this variant is related to severe PE but not mild PE. 

**Table 1:** Genotypic frequency of maternal *NLRP3* polymorphisms in PE and control group

**Polymorphisms**	**Control (N=258)**	**PE (N=252)**	**OR (95% CI)**	**P-value **
**rs10754558**				
CC, n (%)	182 (70.5)	163 (64.7)	1	
CG, n (%)	71 (27.5)	83 (32.9)	1.31 (0.89-1.91)	0.37
GG, n (%)	5 (1.9)	6 (2.4)	1.34 (0.40-4.47)	
				
**rs4612666**				
CC, n (%)	181 (70.2)	161 (63.9)	1	
CT n (%)	67 (26)	75 (29.8)	1.26 (0.85-1.86)	0.23
TT, n (%)	10 (3.9)	16 (6.3)	1.80 (0.79-4.08)	

**Table 2:** The association of the *NLRP3* polymorphisms haplotypes and risk of preeclampsia

**rs10754558/rs4612666**	**Case %**	**Control %**	**OR (95% CI)**	**P-value**
CC	0.738	0.794	1	-
GT	0.139	0.120	1.24 (0.86-1.79)	0.25
CT	0.073	0.048	1.56 (0.92-2.63)	0.097
GC	0.049	0.036	1.50 (0.78-2.89)	0.22

**Figure 1:**
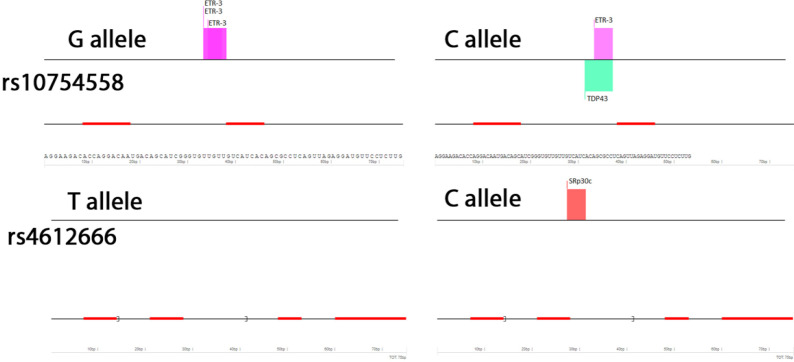
In silico prediction of splice motifs for wild-type and mutated type of *NLRP3* rs10754558 and rs4612666 polymorphisms using SpliceAid 2. The width of the bars depends on the number of nucleotides in the binding region and the height of the bars depends on its affinity (score).

**Figure 2:**
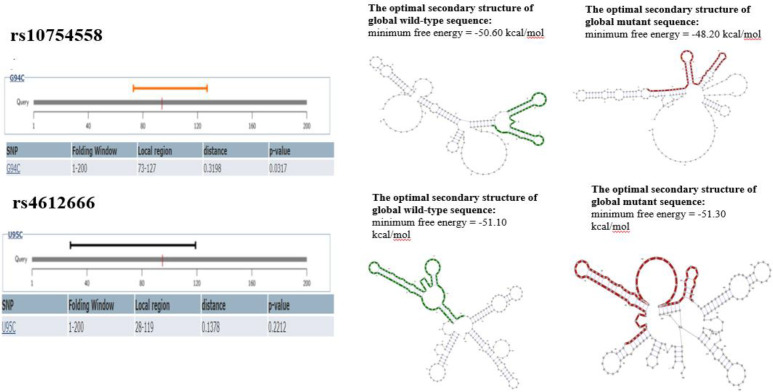
The secondary structure change in RNA due to nucleotide substitution by RNAsnp tool

The SpliceAid 2 tool showed that for the rs10754558 polymorphism in the mutant state, compared to the wild state, two binding sites for ETR-3 protein were lost, and also one site for TDP43 protein was created. This tool for rs4612666 polymorphism showed the creation of a SRp30c protein binding site in the mutant state compared to the wild state (Fig. 1). RNAsnp tool for rs10754558 polymorphism showed a significant secondary structure change in RNA due to nucleotide change, but this secondary structure change was not significant for rs4612666 polymorphism (Fig. 2).

## DISCUSSION

PE is described as the complication of high blood pressure in pregnancy, and also it is among the diseases that can affect the mother and the fetus. Although this complication can remain for the mother after delivery, in most cases, the symptoms disappear after delivery, and this shows the effect of this organ on the pathogenesis of the disease [22]. The defective vascular regeneration system followed by placental ischemia is among the most important causes of PE. In addition, angiogenesis disorders, oxidative stress, angiotensin system imbalance, and exaggerated inflammatory responses can play key roles in the pathogenesis of this disorder [23]. 

Evidence showed a systemic inflammatory response in all pregnancies; however, an extreme intensity in inflammatory responses is observed in pregnancies complicated with PE. Several studies have also indicated the role of quantitative and qualitative modifications in local immune cell responses [5]. A major inflammatory pathway called the inflammasome consists of a multi-protein complex localizing to the intracellular compartment that can activate inflammatory caspase 1 or other inflammatory caspases upon activation [24].

Inflammasomes are the main molecules activating inflammatory responses and playing an important role in the protective response of the immune system to stimuli, such as cellular stress and cell membrane damage which are harmful [6]. This process is firmly regulated; therefore, excessive inflammation is involved in various systemic and chronic inflammatory diseases [7].

NLRP3 is considered as a main protein component of inflammasomes belonging to the Nod-like receptors (NLRs) family. It is expressed in immune cells such as monocytes, neutrophils, dendritic cells, barrier cells, lymphocytes, and neurons and acts as a member of the innate immune system [25]**. **NLRP3 is characterized by its N-terminal pyrin domain (PYD), which enables NLRP3 to employ the ASC as the adaptor molecule via PYD–PYD interactions, thereby facilitating the function of procaspase-1 to build the inflammasome complex [26]. Activated caspase -1 initiates inflammatory reactions via the conversion of pro-IL-18 and pro-IL-1β into their active forms [24]. 

Although activation of the NLRP3 inflammasome can help protect against infections occurring due to microbes, the dysregulation of this pathway has been observed in various diseases [24]. Altered expression of NLRP3 has been observed in various conditions, such as inflammatory diseases and PE [27, 28]. In their study, Weel et al revealed that the IL-1β, TNF-α, Caspase-1, NLRP3, and HMGB1 mRNA expression were increased in the placenta of pregnant women diagnosed with PE [28]. A higher expression level of *NLRP3* in the uterus of preeclamptic rats was observed in Zeng et al.’s study suggesting excessive inflammation at the maternal-fetal interface [29]. 

The association between genetic variants of *NLRP3* and different diseases like PE has been investigated However, the findings were inconsistent. Therefore, in this research, we evaluated the impacts of *NLRP3* variants on PE susceptibility [15, 16]. 

In the present study, no significant relationship was identified between rs10754558 and rs4612666 variants and the PE predisposition. However, a significant relationship was indicated between the rs10754558 variant and severe PE compared to mild PE and controls in several genetic models. Also, a significant relationship was observed between rs10754558 and EOPE compared to the control group only in the dominant model. 

Contrary to our results, Xu et al. found a significant relation between rs10754558 polymorphism and PE in the dominant model. However, similar to our findings, they showed the relationship between this variant and severe PE. Similarly, the *NLRP3* rs2027432 showed no significant effect on PE susceptibility [15]. However, Pontillo et al reported the relationship between *NLRP1* rs12150220 (L155H) and PE development [30]. In their study, Chen et al evaluated the possible impacts of variants in several genes associated with inflammation on PE, and also they reported the relationship between *NLRP3* rs2027432 T and *IL-17RA* rs4819554 G alleles and PE risk [16]**. **Since the severe PE showed the acute form of disease and the early onset PE showed the symptoms earlier. The genetic basis of these condition could be more plausible.

Moreover, several studies reported that there is a relationship between *NLRP3* polymorphisms and inflammatory diseases. In the meta-analysis conducted by Wu et al., the effects of *NLRP3 *rs10754558 and rs35829419 polymorphisms on autoimmune diseases was investigated, and also a significant protective effect of the rs35829419 variant on rheumatoid arthritis (RA) but not overall autoimmune diseases was observed.  In addition, rs10754558 polymorphism significantly decreased the risk of this disease only in Latin Americans. The analysis regarding to disease type indicated the correlation between the G allele of rs10754558 and RA, type 1 diabetes (T1D), and systemic lupus erythematosus (SLE) [31]. In another meta-analysis performed in 2023, there was no relationship between *NLRP3 *polymorphisms (such as rs10754558, rs35829419, and rs4612666) and RA susceptibility [32]. Lee et al. considered the rs10754558 but not the rs35829419 variant as a susceptible factor contributing to SLE and autoimmune and inflammatory diseases only in Latin American individuals [33]. In the study of Zhou et al., the *NLRP3* rs10754558 G variant was associated with coronary artery disease (CAD) and more severe coronary artery stenosis [34].

In exploring the effects of *NLRP3* variants (such as rs4612666 and rs10754558) and T1D, a significant relationship was seen between rs10754558 polymorphism and increased risk of diabetes as well as insulin resistance [35]. Also, there was an association between* NLRP3 *rs10754558 polymorphism and the development of T1D in the Indian population [36]. In another study, there was a relationship between *NLRP3* rs10754558 and the risk of bladder cancer development, lymph node metastasis, the tumor size, mainly in alcohol drinkers and smokers [37]. 

The results obtained from the in silico analysis demonstrated the effects of rs10754558C to G and rs4612666C to T substitutions on protein binding sites. As a result of rs10754558C to G subsituation two binding sites for ETR-3 protein have been lost and also one site for TDP43 protein has been created. In addition for rs4612666 polymorphism the C to T substitution could lead to the generation of a SRp30c protein binding site. The rs10754558C to G substitution showed a significant secondary structure change in RNA due to nucleotide change. These findings could confirm those of previous studies suggesting the effects of these variants on different diseases. Considering the role of these proteins in the processing, stability and translation of RNA [38-40] and the studies conducted on the relationship between these proteins and atherosclerosis diseases [41], neurological disorders [39], systemic lupus erythematosus [42] and various types of cancers [43, 44], the importance of these proteins can be discussed further. 

## Acknowledgment:

This article was extracted from a PhD dissertation at Zahedan University of Medical Sciences (IR.ZAUMS.REC.1399.349). 

## Conflict of Interest:

All authors declare no conflict of interest to disclosure.

## Authors’ Contribution:

 All authors contributed to the study's conception and design. Material preparation, data collection and analysis were performed by MR, MG, RGM, MS, MN and SS. In silico analysis was performed by HSG. The first draft of the manuscript was written by MR and SS. All authors commented on previous versions of the manuscript. All authors read and approved the final manuscript.

## Ethical approval:

This study was performed in line with the principles of the Declaration of Helsinki. The study protocol was approved by the ethics committee of Zahedan University of Medical Sciences (IR.ZAUMS.REC.1399.349).
